# How Reproducible Are Abdominal Wall Surgical Techniques? A Methodological Assessment of Technical Reporting in the Contemporary Literature 

**DOI:** 10.3389/jaws.2026.16371

**Published:** 2026-04-02

**Authors:** Francesco Brucchi, Rita Stanco, Gianlorenzo Dionigi, Filip Muysoms

**Affiliations:** 1 Division of General Surgery, Istituto Auxologico Italiano, IRCCS, Istituto di Ricovero e Cura a Carattere Scientifico, Milan, Italy; 2 Department of Pathophysiology and Transplantation, University of Milan, Milan, Italy; 3 University of Pavia, Pavia, Italy; 4 Department of General Surgery, AZ Maria Middelares, Ghent, Belgium

**Keywords:** abdominal wall surgery, acronyms, hernia repair, methodological study, nomenclature

## Abstract

**Purpose:**

To evaluate the completeness and reproducibility of technical reporting in contemporary abdominal wall hernia literature, and to assess the risk of misinterpretation when surgical techniques are classified based solely on titles and abstracts.

**Methods:**

A descriptive methodological analysis was conducted on original studies published between 2000 and November 2025 reporting abdominal wall hernia repair techniques. The unit of analysis was the surgical technique (surgical arm). Each technique was assessed across five predefined technical domains essential for reproducibility: surgical approach, hernia type, mesh position, mesh type, and fixation method. Techniques were classified as fully reproducible only when all domains were explicitly reported. A secondary analysis evaluated immediate interpretability based on title and abstract information. Terminological variability was explored by identifying distinct acronyms used for identical technical configurations.

**Results:**

Two hundred articles comprising 290 surgical arms were analyzed. Surgical approach and hernia type were almost universally reported (≥99%). In contrast, mesh-related domains were inconsistently described, with mesh position and fixation reported in 81.4% and mesh type in 71.7% of arms. Overall, only 51.7% of techniques were fully reproducible based on full-text assessment. When limited to titles and abstracts, complete interpretability dropped to 3.4%, indicating a high risk of technical misclassification. Substantial terminological redundancy was observed, with up to 16 different acronyms used to describe identical technical configurations.

**Conclusion:**

Technical reporting in abdominal wall surgery is frequently incomplete, substantially limiting reproducibility, evidence synthesis, and reliable interpretation, particularly when relying on titles and abstracts. Excessive and inconsistent use of acronyms further amplifies ambiguity. The strict reliance on explicit reporting may overestimate non-reproducibility in real-world practice, and the study was not designed for exhaustive literature coverage. Adoption of structured, component-based reporting frameworks may represent a pragmatic pathway toward improving clarity, reproducibility, and methodological rigor in abdominal wall surgery.

## Introduction

Abdominal wall surgery encompasses a wide spectrum of techniques applied to heterogeneous defects within a complex anatomical setting, where clinical outcomes are highly dependent on technical execution. Even relatively minor variations in surgical approach, mesh position, material, or fixation may substantially influence postoperative outcomes, complication rates, and recurrence rates [1, 2]. Consequently, detailed and explicit reporting of operative techniques is not merely desirable but essential to ensure reproducibility, meaningful comparison, and accurate interpretation of study results.

Over recent decades, the rapid expansion of surgical techniques has been accompanied by a parallel proliferation of acronyms. Originally intended to facilitate communication and simplify technique identification, their increasing number and heterogeneity have progressively produced the opposite effect. Acronyms are frequently used as substitutes rather than complements to detailed technical descriptions, resulting in publications that omit essential procedural information [3]. Moreover, identical acronyms may be applied to distinct techniques, while the same procedure may be reported under different acronyms, further increasing terminological ambiguity [4].

This variability has important methodological implications. Inadequate or ambiguous technical reporting undermines reproducibility and outcome interpretation and threatens the validity of pooled analyses in systematic reviews and meta-analyses [5]. The problem is further compounded by reliance on titles and abstracts for rapid study appraisal, where critical technical details are often unavailable, increasing the risk of misclassification and inappropriate grouping of surgical techniques.

Against this background, we conducted a methodological study to evaluate the quality and completeness of technical reporting in contemporary abdominal wall hernia literature. By deliberately focusing only on explicitly reported information, this study aims to provide an objective assessment of current reporting practices and to identify structural limitations that may hinder reproducibility and comparability in abdominal wall surgery.

## Materials and Methods

### Study Design

This was an original, descriptive methodological study aimed at evaluating the completeness and reproducibility of surgical technical reporting in contemporary abdominal wall surgery literature.

The study did not assess clinical outcomes, comparative effectiveness, or patient-level data. Its primary objective was to evaluate the completeness of technical reporting based on full-text assessment. A secondary exploratory analysis investigated the immediate interpretability of surgical techniques based solely on information available in titles and abstracts.

The study specifically focused on whether surgical techniques could be unambiguously reconstructed based on explicitly reported information in published articles, without relying on expert inference. Although the study shares some characteristics with a scoping review in terms of breadth and descriptive intent, the unit of analysis was the individual surgical technique rather than the publication, and the research question concerned the internal completeness of technical reporting rather than the mapping of a research field. For these reasons, the study was designed as a descriptive methodological analysis rather than framed within a scoping review framework. The reporting of this study was informed by the guideline for meta-epidemiological methodology research proposed by Murad and Wang [6], adapted to the descriptive nature of the present analysis. No formal protocol was prospectively registered, as protocol registration platforms (e.g., PROSPERO) are designed for systematic reviews of clinical questions and are not applicable to this study type. However, the study design, technical domains, outcome definitions, and analytical framework were all established *a priori* before data extraction.

### Literature Search and Study Selection

A structured literature search was performed in PubMed/MEDLINE to identify original studies reporting surgical techniques for abdominal wall hernia repair. The search covered the period from January 2000 to November 2025 and was last updated in November 2025.

The following search strategy was used, combining free-text terms and MeSH headings:

(“hernia repair” OR “abdominal wall” OR inguinal OR ventral OR incisional OR parastomal) AND (laparoscopic OR robotic OR open OR endoscopic) AND (technique OR approach OR mesh OR repair).

Titles and abstracts were screened independently by two reviewers for relevance. Full-text articles were subsequently assessed for eligibility. Disagreements were resolved by consensus. The screening process was continued until descriptive saturation was reached, defined as the absence of relevant changes in reporting patterns across consecutive batches of studies. Descriptive saturation was operationally defined as the point at which the inclusion of additional consecutive batches of studies (assessed in groups of 20) did not result in meaningful changes in the overall distribution of reporting completeness rates across the five predefined technical domains. This pragmatic threshold was adopted because the objective was not to achieve exhaustive coverage of the literature, but rather to obtain a sufficiently representative and stable estimate of current reporting practices.

Studies were included if they:

- Involved adult patients;
- Addressed abdominal wall hernia repair, including inguinal, ventral, incisional, spigelian, umbilical, or parastomal hernias;
- Reported a defined surgical technique performed using an open, laparoscopic, or robotic approach;
- Were published within the predefined time frame.

Studies were excluded if they:

- Were narrative reviews, systematic reviews, or meta-analyses;
- Were case reports, technical notes without outcome data, or video-only publications;
- Lacked any meaningful technical description of the surgical procedure.

### Unit of Analysis

The unit of analysis was the surgical technique rather than the individual publication.

When a study reported more than one surgical approach or technique, each technique was considered as a separate surgical arm and analyzed independently.

### Definition of Technical Domains

Each surgical arm was evaluated across five predefined technical domains, selected *a priori* as essential elements for reproducibility in abdominal wall surgery:

1. Surgical approach
2. Hernia type
3. Mesh position
4. Mesh type
5. Fixation method

Technical domains were considered reported only when explicitly stated in the manuscript. Information that could be inferred by expert interpretation, contextual assumptions, or visual inspection but was not explicitly described in the text was classified as not reported. These five domains were selected because they represent the minimal set of technical elements required to unambiguously identify a surgical procedure in abdominal wall surgery. Additional factors relevant to surgical outcomes—such as defect size, fascial closure technique, component separation details, and dissection extent—were deliberately excluded from the present analysis. While these elements may influence clinical results, they are not strictly necessary to define the core identity of a surgical technique and would have substantially increased the complexity of the assessment framework.

Surgical techniques sharing identical technical configurations—defined by the combination of the five predefined domains—were identified. For each unique configuration, the number of distinct acronyms or descriptive terms used in the literature was recorded as a descriptive measure of terminological variability. This analysis was exploratory in nature and was not intended as a systematic linguistic assessment.

### Data Extraction and Reviewer Roles

Data extraction was performed using a predefined data dictionary.

Initial data extraction was conducted by a reviewer without formal training in abdominal wall surgery (RS), focusing on the identification of reported technical elements and their location within the manuscript. This approach was adopted to minimize expert-driven interpretation.

A second reviewer with specific expertise in abdominal wall surgery and dedicated fellowship training (FB) independently reviewed all extracted data for accuracy and completeness.

Discrepancies or ambiguous cases were resolved through adjudication by a senior expert in abdominal wall surgery (FM), who provided the final determination when consensus could not be reached.

### Outcomes

The primary outcome was the completeness of technical reporting based on full-text assessment, defined as full reproducibility.

Secondary outcomes included:

- Immediate interpretability of surgical techniques based on title and abstract information;
- Degree of terminological variability across identical technical configurations.

Operational definitions of outcome measures are reported below.

### Definition of Reproducibility

A surgical technique was defined as fully reproducible when all five predefined technical domains were explicitly reported and allowed an unambiguous reconstruction of the procedure.

Techniques for which one or more domains were missing or ambiguously reported were classified as partially reproducible.

### Statistical Analysis

Analyses were descriptive in nature. Results were summarized using absolute counts and percentages.

Exploratory subgroup analyses were performed according to surgical approach and hernia type.

No formal hypothesis testing was planned. As this study did not estimate or pool treatment effects, several items of the Murad and Wang reporting framework for methodology research were not applicable, including risk of bias assessment of individual studies, summary effect measures (e.g., ratio of odds ratios), and evaluation of publication bias. These items are relevant to meta-epidemiological studies that quantify the impact of study-level characteristics on clinical effect estimates, which was not the objective of the present analysis.

### Ethics

This study was based exclusively on published literature and did not involve human participants or individual patient data. Formal ethical approval was therefore not required.

## Results

### Study Sample

**FIGURE 1:**
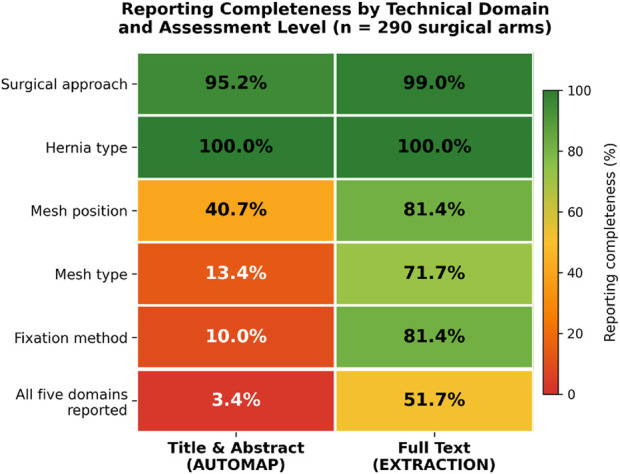
Heatmap illustrating reporting completeness (%) by technical domain and assessment level across 290 surgical arms. Color scale ranges from red (low completeness) to green (high completeness). AUTOMAP = information deducible from title and abstract; EXTRACTION = information explicitly reported in the full text.

The search strategy yielded 23,347 records in PubMed. Titles and abstracts were screened sequentially in chronological batches. Screening was discontinued after descriptive saturation was reached, at which point approximately 467 records had been reviewed. Of these, 467 full-text articles were assessed for eligibility. A total of 267 studies were excluded (narrative or systematic reviews, n = 106; case reports or technical notes, n = 64; insufficient technical description, n = 97), resulting in 200 included articles [7–20], [21–40], [41–60], [61–80], [81–100], [101–120], [121–140], [141–160], [161–180], [181–205]. As several studies reported more than one surgical technique, the unit of analysis was the surgical arm. Overall, 290 distinct surgical arms were evaluated. At the study level, most articles focused on groin (n = 89, 44.3%) and ventral hernias (n = 61, 30.3%), followed by incisional (n = 33, 16.4%), parastomal (n = 11, 5.5%), umbilical (n = 5, 2.5%), and spigelian hernias (n = 2, 1.0%). Study characteristics are summarized in Table 1. Reporting completeness by technical domain is illustrated in Figure 1.

**TABLE 1:** Summary of characteristics of included studies (n = 200).

Characteristic	n (%) or value
Study sample	
Included studies	200
Surgical arms (unit of analysis)	290
Studies with ≥2 arms	81 (40.5%)
Publication period	
2010–2014	59 (29.5%)
2015–2019	76 (38.0%)
2020–2025	65 (32.5%)
Hernia type (study level)	
Groin	89 (44.5%)
Ventral (primary or mixed)	60 (30.0%)
Incisional	33 (16.5%)
Parastomal	11 (5.5%)
Umbilical	5 (2.5%)
Spigelian/Lumbar/Other	2 (1.0%)
Surgical approach (study level, predominant)	
Open	49 (24.5%)
Laparoscopic	47 (23.5%)
Endoscopic extraperitoneal	39 (19.5%)
Robotic	7 (3.5%)
Comparative (multiple approaches)	58 (29.0%)
Geographic region	
Europe	80 (40.0%)
Asia–Pacific	46 (23.0%)
North America	36 (18.0%)
Middle East/Western Asia	15 (7.5%)
Latin America	12 (6.0%)
Africa	6 (3.0%)
Other	5 (2.5%)
Journal (top 5)	
Hernia	62 (31.0%)
Surgical Endoscopy	47 (23.5%)
World journal of surgery	9 (4.5%)
JSLS	6 (3.0%)
Other (39 journals)	76 (38.0%)

Values are presented as n (%) unless otherwise specified. Study-level classification was based on the predominant hernia type and surgical approach. In comparative studies reporting multiple approaches, each arm was classified independently in the main analysis.

### Completeness of Reporting Based on Full-Text Assessment

Based on explicit information reported in the full text, surgical approach and hernia type were almost universally described (99.0% and 100%, respectively).

In contrast, mesh-related variables were less consistently reported. Mesh position and fixation method were explicitly reported in 81.4% of arms, while mesh type was reported in 71.7%.

When considering all five technical domains together, only 150 of 290 arms (51.7%) met the criteria for full reproducibility, defined as explicit reporting of surgical approach, hernia type, mesh position, mesh type, and fixation method.

### Immediate Interpretability From Title and Abstract

When analysis was limited to information deducible from titles and abstracts (AUTOMAP), reporting completeness was substantially lower.

While surgical approach and hernia type were frequently identifiable (95.2% and 100%, respectively), mesh-related information was rarely deducible without full-text review. Mesh position was identifiable in 40.7% of arms, mesh type in 13.4%, and fixation method in only 10.0%.

As a result, only 10 of 290 surgical arms (3.4%) were fully interpretable based on title and abstract alone.

### Terminological Variability and Acronym Redundancy

Using the acronym dictionary, technical configurations were defined based on the combination of surgical approach, hernia type, and mesh position, excluding only entries in which any of these three domains was unspecified. This resulted in a set of clearly defined technical configurations suitable for descriptive analysis.

When grouped according to these criteria, substantial terminological variability was observed. Multiple distinct acronyms or descriptive terms were frequently used to denote identical technical configurations, despite the absence of meaningful procedural differences.

The highest degree of redundancy was identified for a single configuration—open approach, incisional hernia, and retromuscular mesh position—which was described using 16 different acronyms or terms across the literature. These denominations included both formal acronyms and narrative labels referring to the same underlying surgical construct.

Overall, this analysis demonstrates that even when focusing on a limited number of fundamental technical domains, considerable heterogeneity in terminology persists. Representative examples of terminological redundancy across identical technical configurations are provided in Supplementary Table S1.

## Discussion

In this methodological analysis of contemporary abdominal wall surgery literature, we found that while basic procedural descriptors such as surgical approach and hernia type are almost universally reported, critical elements required for technical reproducibility—namely mesh position, mesh type, and fixation method—are frequently omitted. Even after full-text assessment, only approximately half of the analyzed surgical techniques could be considered fully reproducible based on explicitly reported information.

This limitation was even more pronounced when evaluating immediate interpretability based solely on titles and abstracts, where fewer than 5% of techniques could be unambiguously reconstructed. Together, these findings reveal a substantial discrepancy between how surgical techniques are named or labeled and how completely they are described, pointing to a structural weakness in current reporting practices.

The proliferation of acronyms in abdominal wall surgery has been previously documented. Ramana et al. showed that the rapid expansion of minimally invasive and reconstructive techniques has been accompanied by an uncontrolled growth of acronyms, often inconsistently applied and poorly standardized [206]. Although acronyms are intended to facilitate communication, their excessive and heterogeneous use may paradoxically increase ambiguity rather than reduce it. Consistently with this interpretation, we observed that identical technical configurations were frequently described using multiple distinct acronyms. In our dataset, more than one acronym was used for the same technical configuration in over one quarter of cases, introducing terminological heterogeneity unrelated to actual procedural differences.

Our findings extend this observation from a primarily semantic concern to a methodological one. Rather than functioning as reliable descriptors of surgical technique, acronyms frequently act as shorthand labels that convey only partial or superficial information. As demonstrated in our analysis, acronyms rarely encapsulate the technical elements required for reproducibility and, in some cases, may even obscure them. Moreover, identical techniques may be described using different acronyms, while the same acronym may refer to distinct procedures, further complicating literature interpretation and evidence synthesis.

The consequences of imprecise or incomplete technical terminology in abdominal wall surgery have been highlighted previously. Parker and colleagues demonstrated that inconsistent use of terms describing mesh planes has led to substantial misclassification in the literature, including inappropriate pooling of heterogeneous techniques in systematic reviews and meta-analyses [5]. Our results build on these observations by showing that, beyond inconsistent terminology, essential technical information is frequently not explicitly reported at all, even in full-text manuscripts. In this context, ambiguity arises not only from misuse of terms, but also from omission of key procedural details.

Clear and reproducible reporting of surgical techniques is a prerequisite for meaningful comparison across studies, meta-analyses, and guideline development. As emphasized by Muysoms and Vierstraete, ambiguity in technique description hampers the ability to aggregate data and interpret outcomes reliably. Our empirical findings support this concern, demonstrating that reliance on acronyms or brief narrative descriptions—without structured technical detail—substantially limits reproducibility [4].

The recently proposed Muysoms nomenclature offers a rationalized approach to surgical technique description by decomposing each procedure into five fundamental components: approach, hernia type, mesh position, mesh type, and fixation [3, 4]. Conceptually, this modular framework directly addresses the deficiencies identified in our analysis by shifting the focus away from names and acronyms toward explicitly reported technical domains.

Although our study was not designed to validate or compare classification systems, the close alignment between the domains most frequently underreported in the literature and those explicitly encoded in the Muysoms framework is notable. This suggests that structured, component-based nomenclature may represent a pragmatic pathway toward improving clarity, reproducibility, and machine-readability of surgical reporting.

From a practical perspective, incomplete technical reporting has direct implications for evidence synthesis, guideline development, and registry-based research. Inadequately described techniques increase the risk of inappropriate pooling in systematic reviews, limit the interpretability of registry data, and hamper secondary analyses based on large datasets. Moreover, the lack of standardized, machine-readable technical descriptors constrains the development of automated literature screening and artificial intelligence–based evidence synthesis tools.

This study has several limitations. The analysis was restricted to PubMed-indexed literature and deliberately relied on explicit reporting rather than expert inference, which may underestimate the practical understanding of experienced surgeons. However, this approach was intentional, as the objective was to assess objective reproducibility rather than subjective interpretability. Furthermore, the strict reliance on explicit reporting may overestimate the degree of non-reproducibility in real-world practice, as experienced surgeons can often infer missing technical details from contextual cues or institutional conventions. In addition, the number of included studies was determined by descriptive saturation rather than exhaustive inclusion. The broad inclusion period (2000–2025) may have introduced temporal heterogeneity, as both terminology and reporting standards have evolved over time. While this wide timeframe was chosen to reflect the full spectrum of contemporary surgical literature, it is possible that more recently published studies may exhibit more complete reporting than earlier ones. Formal temporal subgroup analyses were not performed, as the study was not powered for this purpose. Future studies with larger, systematically sampled datasets should investigate temporal trends in reporting completeness. In addition, potential predictors of reporting completeness—such as journal type, impact factor, study design, and publication year—were not formally assessed. It is plausible that journals with more rigorous editorial standards or structured reporting requirements may achieve higher completeness rates. Exploratory analyses investigating these factors were beyond the scope of the present study, which was primarily designed to quantify reporting completeness rather than to identify its determinants. Furthermore, the assessment was deliberately focused on five fundamental technical domains; other elements relevant to surgical outcomes—such as defect size, fascial closure technique, component separation details, and dissection extent—were not evaluated. While these factors may influence clinical results, they are not strictly necessary to define the core identity of a surgical technique and would have substantially increased the complexity of the assessment framework.

Future efforts should focus on evaluating structured reporting tools and nomenclature systems in larger datasets, across registries and guideline documents, and on exploring their integration into editorial and reporting standards. Improving technical reporting is therefore not merely a semantic exercise. It represents a necessary prerequisite for reliable evidence synthesis, meaningful comparison of surgical outcomes, and the development of transparent, data-driven standards in abdominal wall surgery.

## Data Availability

The original contributions presented in the study are included in the article/Supplementary Material, further inquiries can be directed to the corresponding author.
