# Supplementary material for: How Reproducible Are Abdominal Wall Surgical Techniques? A Methodological Assessment of Technical Reporting in the Contemporary Literature
Source: J Abdom Wall Surg. 2026 Apr 2;5:16371. doi: 10.3389/jaws.2026.16371 (PMC13083044; doi:10.3389/jaws.2026.16371)
Supplement: Supplementary file 1 [file Table1.docx]

## Supplementary Table S1. Examples of terminological redundancy across identical technical configurations

The table reports representative examples in which identical technical configurations, defined by surgical approach, hernia type, and mesh position, were described using multiple distinct acronyms or descriptive terms in the literature. This table is illustrative and not intended as a systematic analysis of all acronyms.

| Technical configuration* | Acronyms / terms used in the literature |
| --- | --- |
| Open approach; incisional hernia; retromuscular mesh position | LIH repair; open repair; open retromuscular mesh repair; open retromuscular LIH repair; SIH hernia open surgical repair; RSR; OIHR; open Rives retromuscular repair; RM mesh technique; MPFH; open IHR; PCS-TAR; open incisional hernia repair; incisional hernia repair; sublay technique; Rives–Stoppa repair |
| Endoscopic approach; inguinal hernia; preperitoneal mesh position | TEP; eTEP; endoscopic preperitoneal repair; totally extraperitoneal repair |
| Laparoscopic approach; inguinal hernia; preperitoneal mesh position | TAPP; laparoscopic preperitoneal repair; transabdominal preperitoneal repair |
| Robotic-assisted approach; ventral hernia; retromuscular mesh position | rTARUP; rTARM; robotic retromuscular repair |

*Technical configuration defined by the combination of surgical approach, hernia type, and mesh position. Entries were excluded only when one of these three domains was unspecified.
